# Mammillary body regulates state-dependent fear by alternating cortical oscillations

**DOI:** 10.1038/s41598-018-31622-z

**Published:** 2018-09-07

**Authors:** Jun Jiang, Guang-Yu Wang, Wenhan Luo, Hong Xie, Ji-Song Guan

**Affiliations:** 1 School of Life Sciences, Tsinghua University, Beijing, 100086 China; 2 Peking-Tsinghua Center for Life Sciences, Beijing, 100871 China; 3 School of Life Science and Technology, ShanghaiTech University, Shanghai, 201210 China; 4 Academy for Advanced Interdisciplinary Studies, Peking University, Beijing, 100871 China; 5 Institute of Brain-intelligence Science and Technology, Zhangjiang Lab, Shanghai, 200031 China; 6 CAS Center for Excellence in Brain Science and Intelligence Technology, Chinese Academy of Sciences, Shanghai, 200031 China

## Abstract

State-dependent memory describes a phenomenon that memory will be efficiently retrieved only when the brain state during retrieval matches the state during encoding. While a variety of psychoactive drugs, such as ethanol, cocaine, morphine and NMDA receptor antagonists, are able to induce state-dependent memory, the biological hallmark of brain state and neural mechanism of its regulation are still unknown. In this study, we found that MK-801 enhanced delta oscillations in awake mice, representing a drug-induced brain state, in which fear memory could only be successfully retrieved when the same drug condition was presented. We identified a key nucleus, mammillary body (MB), which regulates the specific brain state associated with MK-801. Chemogenetic silencing of MB neurons enhanced cortical delta oscillations and generated state-dependent memory. Moreover, optogenetic reconstitution of delta oscillations alone facilitated retrieval of fear memory encoded under MK-801. Our results indicated that delta oscillations in awake animals defined a specific brain state, in which memory formed is inaccessible under the normal condition, shining light on the neural mechanism underlying the fluctuation of memory retrieval and the role of MB in memory encoding and recall.

## Introduction

Memory of past experience and knowledge helps us escape from danger and enables us to make better choice in the future. However, successful recall of memory is not always a persistent phenomenon. Apart from the external environment and context, memory retrieval also depends on the internal brain state, termed as state-dependent memory. State-dependent learning or state-dependent memory describes a phenomenon, if memory is formed when the subject is under the influence of certain mood, pain, emotion or centrally acting drug, it will be thereafter recalled most efficiently only when the same mental or drug condition is reestablished^1–6^. Alcohol is one of the most commonly reported drugs, people can recall events that occurred during drinking episodes only when he is drunk again. Anxiolytic, anesthetic, antipsychotic, narcotic, and stimulant drugs can induce state-dependent memory^2^. Such state-dependent-amnesia phenomenon also appears in somnambulism and in multiple personality syndrome^7,8^. Thus, restricted access to memory not only affects daily performance but also may increase the risk of severe psychiatric disorders. Although this phenomenon has been reported centuries ago^9^, most of the research focuses on the pharmacological induction of state-dependent memory^2–4,10^, leaving its neural mechanism largely unknown.

Natural brain states, including awake and sleep, are different in brain oscillations. Network oscillatory activity reflects the dynamic interaction and synchrony of neural ensemble for information coding^11^. For example, while theta rhythm is a dominant pattern of local field potential (LFP) during voluntary behavior as well as rapid-eye-movement (REM) sleep^12,13^, delta oscillations are the hallmark of deep sleep, contributing to memory consolidation^14,15^. Moreover, recent study has also shown that GABA_A_ receptor agonist gaboxadol, a previously proved state-dependent learning inducing drug, significantly enhances hippocampal delta power^5,16^, suggesting brain oscillations may be associated with the phenomenon of state-dependent memory.

In this study, using the mouse model of contextual fear conditioning, we found that the amnesia induced by a low dose of MK-801, a non-competitive NMDA receptor blocker, actually is a phenomenon of state-dependent memory mediated by inhibition of mammillary body (MB). Both MK-801 and inhibition of MB neurons generated an alternative state, in which delta waves dominated cortical oscillations in awake mice. Moreover, we found that artificially enhancing delta oscillations was able to induce state-dependent fear memory, and the memory encoded under MK-801 could be retrieved by reconstruction of cortical delta oscillations. Our findings suggest that MB is an important regulatory nucleus for brain state and mediates state-dependent fear induced by MK-801 through governing the rhythm pattern of neocortex.

## Results

### Low-dose MK-801 induced amnesia is a phenomenon of state-dependent memory

**Figure 1:**
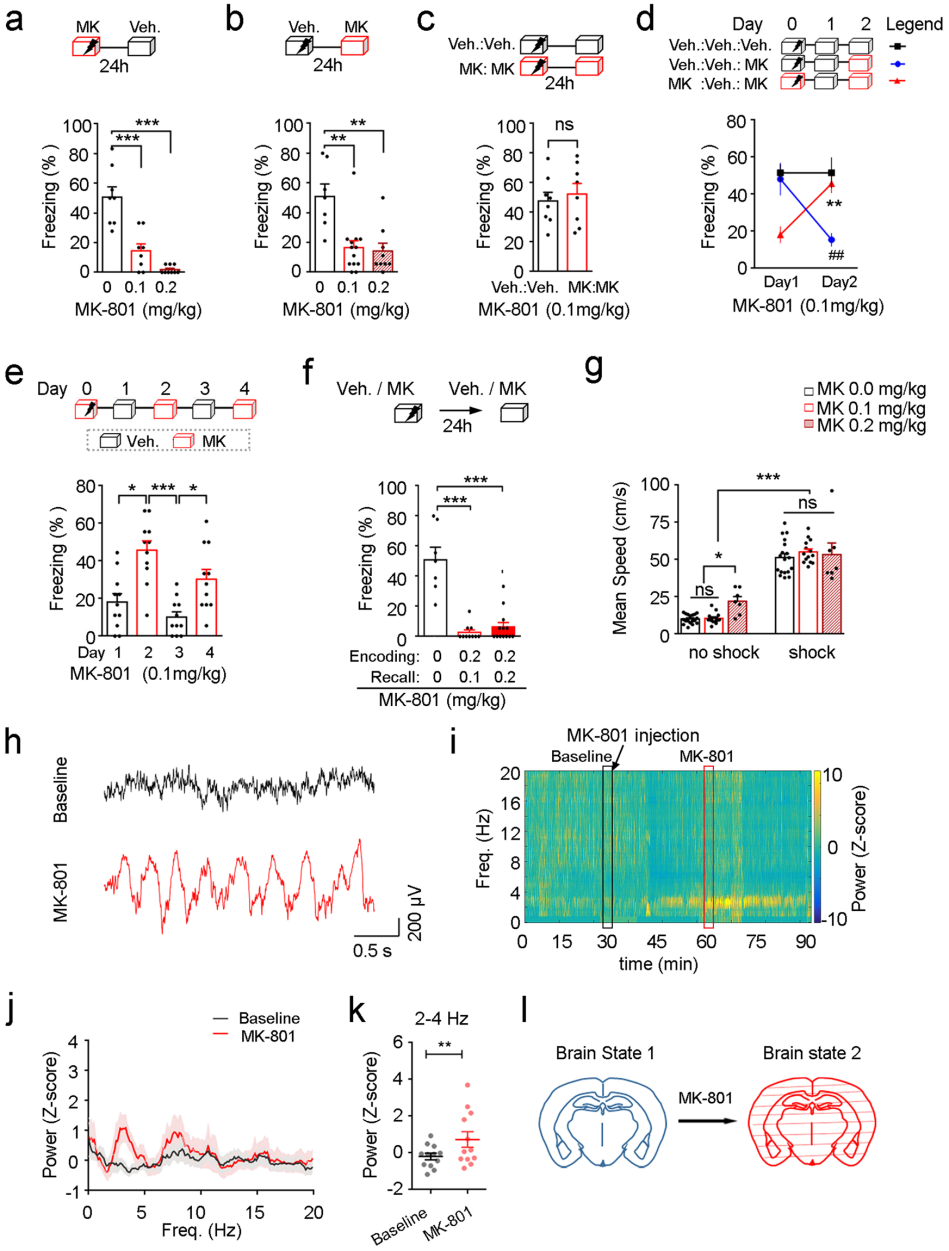
MK-801 induces state-dependent fear memory and enhances cortical delta oscillations. (**a**) Effect of different doses of MK-801 injected (i.p.) 30 min before fear conditioning on freezing (*n* = 8, 8, 9 mice, one-way ANOVA followed by Tukey’s test). (**b**) Effect of different doses of MK-801 injected (i.p.) 30 min before test on freezing (*n* = 7, 13, 9 mice, one-way ANOVA followed by Tukey’s test). (**c**) Effect of MK-801 injected (0.1 mg/kg) both before conditioning and before test on freezing (*n* = 8, 8 mice, unpaired *t* test). (**d**) Mice injected with vehicle/MK-801 before training only froze when tested under the same vehicle/MK-801 condition (*n* = 7, 9, 11 mice, two-way ANOVA followed by Sidak’s test). (**e**) Mice trained with MK-801 (0.1 mg/kg) showed robust freezing when tested with MK-801 (*n* = 11 mice, one-way ANOVA followed by Tukey’s test). (**f**) MK-801 when injected at the dose of 0.2 mg/kg impaired fear memory in mice (*n* = 7, 10, 13 mice, one-way ANOVA followed by Tukey’s test). (**g**) Mean speed measured without or with foot-shock after the injection of MK-801 (i.p.) at different doses (*n* = 18, 14, 7 mice, two-way ANOVA followed by Tukey’s test). (**h**) Representative raw traces of cortical LFPs under the basal (black) and the MK-801 (red) conditions. (**i**) One example of the temporal/spectral structures of cortical LFPs under the influence of MK-801 (0.1 mg/kg, i.p.). Black/red box (5 min) labels the signal used as baseline/MK-801 onboard in the following statistical analysis. (**j**) Average power spectrum of the baseline and the MK-801 onboard periods (*n* = 12 sites from 7 mice). Shadow areas indicate SEM. (**k**) Quantification of the average power of delta band (2–4 Hz) during the baseline and the MK-801 onboard periods (*n* = 12 sites from 7 mice, paired *t* test). (**l**) Illustration of MK-801 induced brain state transitions. ns: *P* > 0.05, *P < 0.05, **P < 0.01, ***P < 0.001, ^##^*P* < 0.01. Veh., vehicle. MK, MK-801. Data presented as mean ± SEM.

Our discovery of the state-dependent fear memory induced by MK-801 was raised from an experiment, originally designed to test the effect of MK-801 on memory retrieval. MK-801 is a non-competitive antagonist of NMDA receptor which is the molecular basis of learning and memory^17–19^. We first examined the effect of MK-801 on memory encoding and recall. MK-801 was injected either before training or before test (Fig. 1a,b), both of which impaired freezing (Fig. 1a, *P* < 0.001, 0 mg/kg vs. 0.1 mg/kg, *P* < 0.001, 0 mg/kg vs. 0.2 mg/kg, *n* = 8, 8, 9 mice, one-way ANOVA followed by Tukey’s test, Fig. 1b, P < 0.01, 0 mg/kg vs. 0.1 mg/kg, *P* < 0.01, 0 mg/kg vs. 0.2 mg/kg, *n* = 7, 13, 9 mice, one-way ANOVA followed by Tukey’s test). These results could be interpreted either as impaired learning or impaired memory retrieval by MK-801. However, when mice were injected with MK-801 (0.1 mg/kg) both before training and before test (MK-MK group), they showed robust freezing (Fig. 1c, *t*_14_ = 0.5050, *P* = 0.6214, *n* = 8, 8 mice, unpaired *t* test). This effect was replicated in a within-subject study, when mice trained with vehicle or MK-801 and then tested with or without drug on alternate tests (Fig. 1d, *P* < 0.01, Group 2 Day 1 vs. Group 2 Day 2, *P* < 0.01, Group 3 Day 1 vs. Group 3 Day 2, *n* = 7, 9, 11 mice, two-way ANOVA followed by Sidak’s test). Furthermore, fear memory encoded in the presence of MK-801 could be successfully recalled in multiple trials under MK-801, but not in trials injected with vehicle (Fig. 1e, *P* < 0.05, Day 1 vs. Day 2, *P* < 0.001, Day 2 vs. Day 3, *P* < 0.05, Day 3 vs. Day 4, *n* = 11 mice, one-way ANOVA followed by Tukey’s test). On the contrary, higher-dose MK-801 (0.2 mg/kg) injected before training totally blocked memory acquisition (Fig. 1f, *F*_2, 27_ = 34.58, *P* < 0.001, *n* = 7, 10, 13, one-way ANOVA followed by Tukey’s test). Though higher dose of MK-801 has dissociative anesthetic effect on the central nervous system^20,21^ and induce hyperactive-locomotion activities (Fig. 1g, *P* < 0.05, no shock 0 mg/kg vs. no shock 0.2 mg/kg, *n* = 18, 7 mice, two-way ANOVA followed by Tukey’s test), the lower dose (0.1 mg/kg) we used here did not affect locomotor activity and foot-shock induced responses (Fig. 1g, *P* > 0.05, no shock 0 mg/kg vs. no shock 0.1 mg/kg, *P* > 0.05, shock 0 mg/kg vs. shock 0.1 mg/kg, *n* = 18, 14 mice, two-way ANOVA followed by Tukey’s test). Thereby, these data suggested that MK-801 at the lower dose (0.1 mg/kg) neither impaired memory encoding nor memory recall, but induced state-dependent fear memory.

### MK-801 induces delta oscillations in neocortex which is a hallmark of the alternative state

Brain state has been characterized as cortical oscillations^22–24^ which coordinate cortical communications and contribute to the acquisition and long-term consolidation of memory^25–27^. Next, we investigated if MK-801 altered the brain state for state-dependent memory by changing cortical oscillatory rhythm. To this end, we recorded local field potential (LFP) signals in head-fixed mice under MK-801 (0.1 mg/kg, i.p.). Surprisingly, in awake animals, MK-801 significantly increased the amplitude of cortical delta waves, which is normally observed during deep sleep^28^ (Fig. 1h–k, *t*_11_ = 3.417, *P* = 0.0058, *n* = 12 sites from 7 mice, paired *t* test, ACC and RSC). The same phenomenon was also observed in freely moving animals (data not shown). Thus, MK-801 induced a distinct oscillatory rhythm which may contribute to the state-dependency of memory encoding and recall (Fig. 1l).

### MK-801 administration suppresses the neuronal activity of the mammillary body

**Figure 2:**
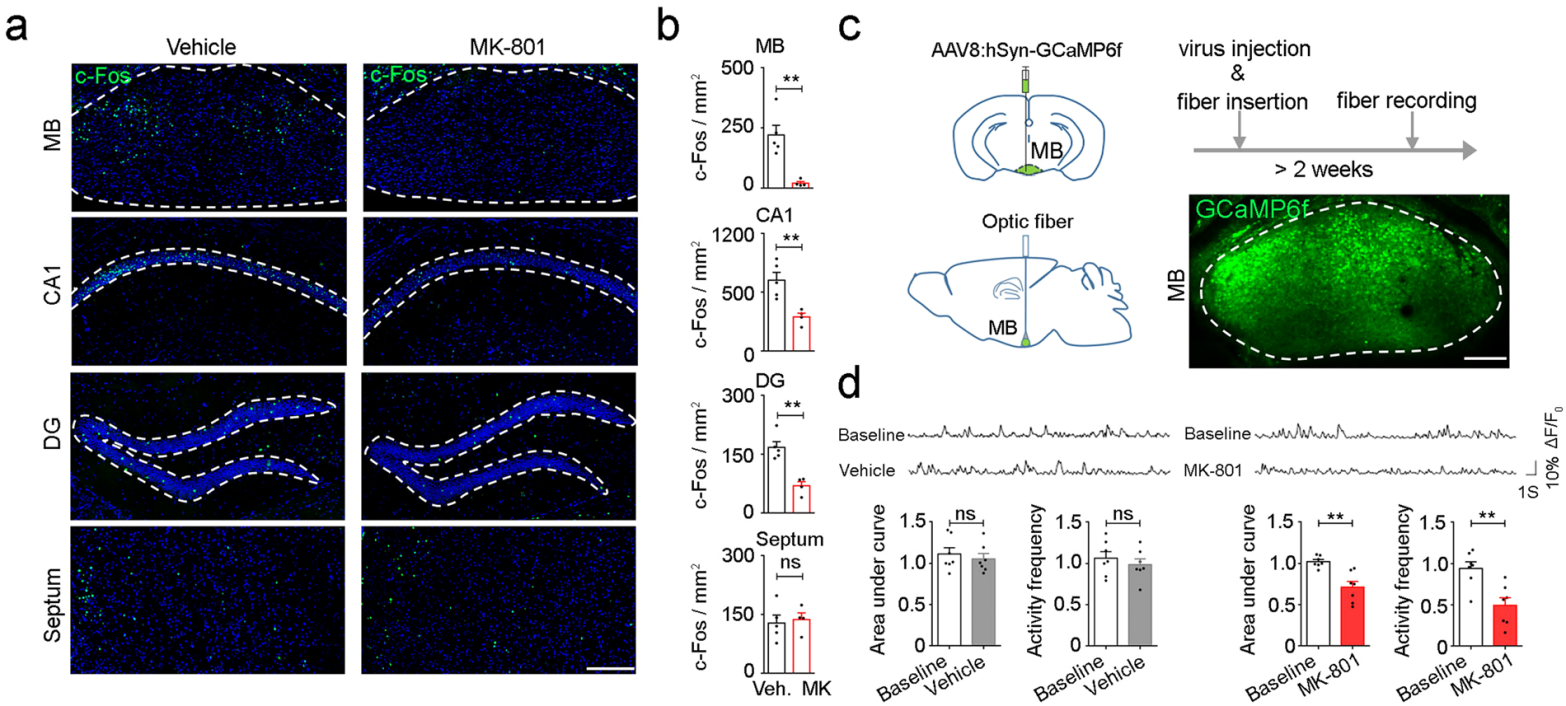
Activity of the mammillary body is repressed upon MK-801 administration. (**a**) Representative images of c-Fos immunostaining in the MB, CA1, DG and septum under the vehicle and the MK-801 (i.p., 0.1 mg/kg) conditions. Green, c-Fos. Blue, DAPI. Scale bar, 200 μm. (**b**) MK-801 reduced the expression of c-Fos in the MB, CA1 and DG, but not septum (*n* = 5, 4 mice, unpaired *t* test). (**c**) Left, schema showing injection of AAV8: hSyn-GCaMP6f and subsequent fiber implantation into the MB. Right bottom, one example image of virus expression in the MB. Green, GCaMP6f. Scale bar, 200 μm. (**d**) Top, representative traces of calcium transients in the MB before and after (30 min later) vehicle (left) or MK-801(right) injection. Bottom left: injection of vehicle had no effect on the basal activity in the MB. Bottom right: MK-801 reduced both the amplitude and the frequency of the calcium transients in the MB (*n* = 7 from 5 mice each, paired *t* test) (Each point represents a normalized average signal of 5 min. Time of injection: 0 min; baseline: - 5 to 0 min; vehicle/MK-801: 30 to 35 min). ns: *P* > 0.05, **P < 0.01. Veh., vehicle. MK, MK-801. Data presented as mean ± SEM.

Previous studies of the expression of immediate early genes (e.g., zif268 and c-Fos) have indicated the involvement of different brain circuits in the recall of recent and remote memory^29,30^. To map the neural circuit underlying the state change caused by MK-801, we examined c-Fos expression across the entire brain under the vehicle and MK-801 conditions. c-Fos expression was reduced in most of the brain regions including both cortical (Supplementary Fig. 1a) and subcortical areas (Fig. 2a,b) upon MK-801 injection. Surprisingly, we found that besides the hippocampus (CA1, *t*_7_ = 3.855, *P* = 0.0063, *n* = 5, 4 mice, DG, *t*_7_ = 5.075, *P* = 0.0014, *n* = 5, 4 mice, unpaired *t* test), MK-801 strongly reduced c-Fos expression in the mammillary body (MB) (*t*_7_ = 4.408, *P = *0.0031, *n* = 5, 4 mice, unpaired *t* test), indicating MB might be a potential target of MK-801 inducing state-dependent memory. In contrast, the septum showed no activity change in response to MK-801 (*t*_7_ = 0.3225, *P* = 0.7565, *n* = 5, 4 mice, unpaired *t* test). In consistent with the dramatic inhibitory effect of MK-801 on MB neurons, we found that the expression level of NR1, a ubiquitously expressed NMDA receptor subunit^31^, is significantly higher in the MB compared to other areas (Supplementary Fig. 1b–d, *P* < 0.01, Sep vs. MB, *P* < 0.05, MB vs. Hip, *P* < 0.05, MB vs. Cortex, *n* = 8 from 4 mice, technical repeat: 2, one-way ANOVA followed by Tukey’s test), suggesting MB as one of the major target of MK-801 in the brain.

To directly evaluate MB neuronal activity under MK-801 *in vivo*, we injected adeno-associated virus (AAV8) expressing GCaMP6f into MB and monitored its calcium transients in freely moving mice with fiber photometry recording^32^ (Fig. 2c). While injection of vehicle had no effect on the basal neuronal activity of MB (Fig. 2d left, *t*_6_ = 1.742, *P* = 0.1321, area under curve, *t*_6_ = 0.6619, *P* = 0.5326, activity frequency, *n* = 7 from 5 mice, paired *t* test), MK-801 significantly reduced both the amplitude and frequency of calcium transients in MB after injection (Fig. 2d right, *t*_6_ = 4.152, *P* = 0.0060, area under curve, *t*_6_ = 3.918, *P* = 0.0078, activity frequency, *n* = 7 from 5 mice, paired *t* test), confirming the effect of MK-801 on suppressing MB neuronal activity in freely moving animals.

MB is known to be an essential regulatory nucleus for learning and memory. MB atrophy, usually resulted from excessive alcohol consumption and thiamine deficiency, has been demonstrated to account for the memory dysfunction in patients with Korsakoff syndrome^33–35^. Pyrithiamine-induced thiamine deficiency (PTD) treatment in rats produced histologic lesions of MB and impaired memory, both of which could be protected by MK-801^20,21,36^. Combined with our data, we speculated that MB may be associated with MK-801 generated state-dependent memory.

### Mammillary body mediates MK-801 induced state-dependent fear memory

**Figure 3:**
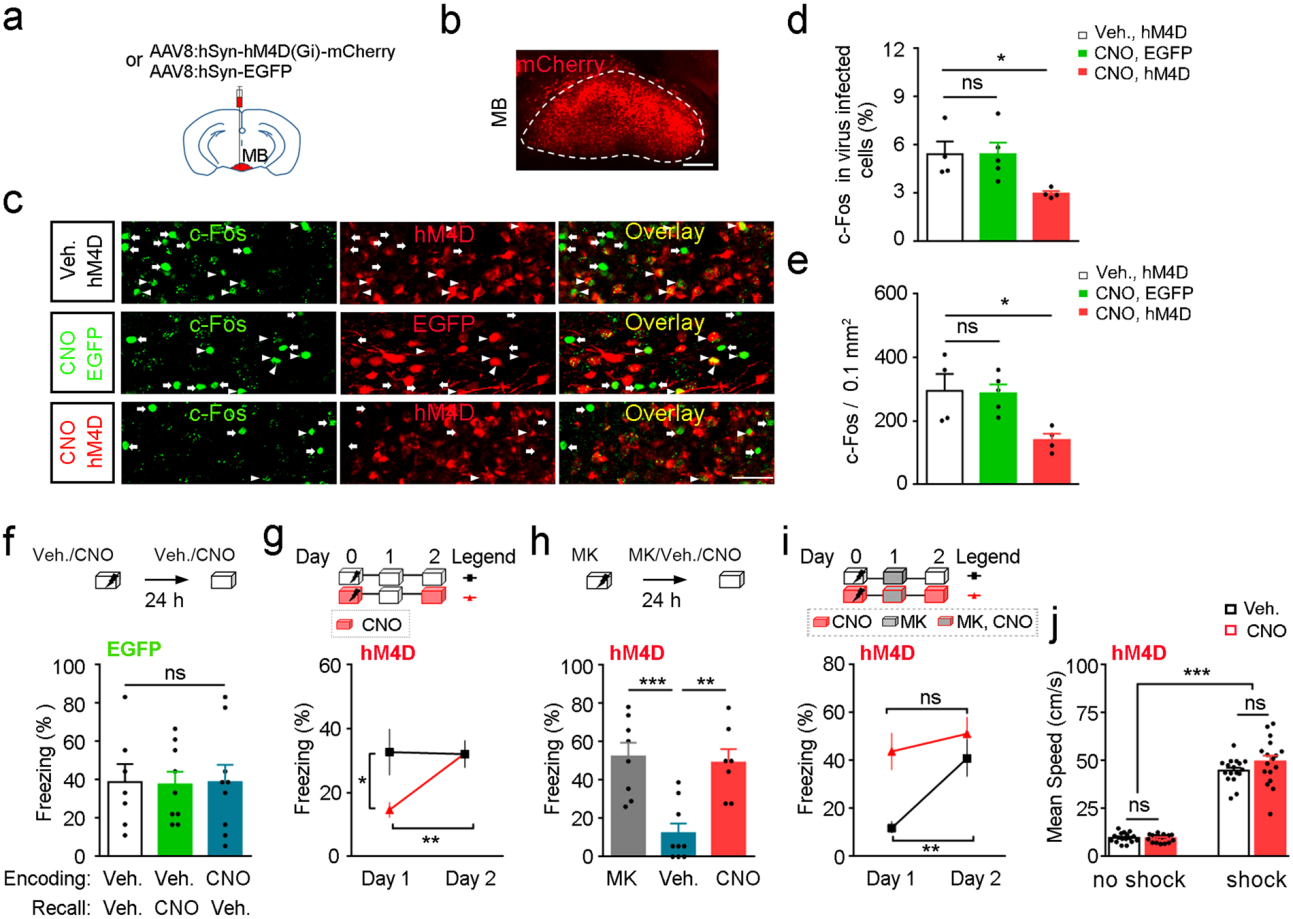
Mammillary body mediates MK-801 induced state-dependent fear. (**a**) Schema for chemogenetic inhibition of MB. (**b**) Representative image of virus expression in the MB. Red, hM4D-mCherry. Scale bar, 200 μm. (**c**) Representative images showed that CNO reduced c-Fos level in the MB expressing hM4D while not that injected with the control virus. Mice injected with either vehicle or CNO (2 mg/kg) in their homecages were perfused 2 hours later. Brains were processed for c-Fos analysis. Arrows label the c-Fos+ virus- neurons and arrow heads label the c-Fos+ virus+ neurons in the MB. Green, c-Fos. Red, hM4D/EGFP. Scale bar, 50 μm. (**d**) CNO reduced the percentage of c-Fos-positive neurons in the hM4D-expressing MB cells (*n* = 4, 5, 4 mice, one-way ANOVA followed by Tukey’s test). (**e**) CNO reduced c-Fos level in MB expressing hM4D (*n* = 4, 5, 4 mice, one-way ANOVA followed by Tukey’s test). (**f**) CNO administration either before training or before test had no effect on the fear memory in the control group (*n* = 7, 9, 9 mice, one-way ANOVA followed by Tukey’s test). (**g**) Mice expressing hM4D in the MB injected CNO before training only froze when tested under CNO (*n* = 9, 11 mice, two-way ANOVA followed by Sidak’s test). (**h**) Mice injected MK-801 before training showed robust freezing when tested either under MK-801 or in the MB-inhibition state but not in the vehicle-injection condition (*n* = 8, 9, 7 mice, one-way ANOVA followed by Tukey’s test). (**i**) MK-801 injection before test significantly reduced the freezing level of mice trained in the normal condition. Whereas, MK-801 injection before test in mice both trained and tested under the MB inhibition condition had no effect on fear behavior (*n* = 8, 9 mice, two-way ANOVA followed by Sidak’s test). (**j**) Chemogenetic inhibition of MB had no effect on the basal locomotion and foot-shock-induced hyperactivity in mice (*n* = 17 mice per group, two-way ANOVA followed by Sidak’s test). ns: *P* > 0.05, *P < 0.05, **P < 0.01, ***P < 0.001. Veh., vehicle. MK, MK-801. Data presented as mean ± SEM.

To test if MB is involved in state-dependent memory, we applied chemogenetic silencer hM4D, the repressive form of designer receptor exclusively activated by designer drugs (DREADD)^37^ to artificially suppress the neural activity of MB (Fig. 3a–e). In mice with MB neurons expressing hM4D, Clozapine-*N*-oxide (CNO, 2 mg/kg, i.p.) reduced c-Fos expression in the virus infected MB neurons (Fig. 3d, *P* < 0.05, Veh. hM4D vs. CNO hM4D, *n* = 4, 4 mice, one-way ANOVA followed by Tukey’s test), and the overall c-Fos level was also suppressed (Fig. 3e, *P* < 0.05, Veh. hM4D vs. CNO hM4D, *n* = 4, 4 mice, one-way ANOVA followed by Tukey’s test), suggesting that there may exist feedforward connections among MB neurons. In contrast, CNO had no effect on c-Fos expression in MB infected with the control virus (Fig. 3d, *P* > 0.05, Veh. hM4D vs. CNO EGFP, *n* = 4, 5 mice, one-way ANOVA followed by Tukey’s test; Fig. 3e, *P* > 0.05, Veh. hM4D vs. CNO EGFP, *n* = 4, 5 mice, one-way ANOVA followed by Tukey’s test). While CNO had no effect on the encoding and recall of fear memory in mice injected the control virus into MB (Fig. 3f, *F*_2, 22_ = 0.007566, *P* = 0.9925, *n* = 7, 9, 9 mice, one-way ANOVA followed by Tukey’s test), mice expressing hM4D in MB neurons trained with CNO showed significantly reduced fear response to the conditioning context in the test trial without CNO (Fig. 3g, *P* < 0.05, Day 1 Group 1 vs. Day 1 Group 2, *n* = 9, 11 mice, two-way ANOVA followed by Sidak’s test). The same group of mice showed robust freezing when tested with CNO (Fig. 3g, *P* < 0.01, Day 1 Group 2 vs. Day 2 Group 2, *n* = 11 mice, two-way ANOVA followed by Sidak’s test), indicating that memory encoded in the MB inhibition state could be efficiently retrieved when the same state was reestablished.

To further explore whether MB neuronal activity is involved in the state-dependent fear induced by MK-801, mice were trained with MK-801 and fear memory was tested under different states (Fig. 3h). We found mice with repressed MB activity during test showed comparable freezing to mice tested with MK-801 and was significantly higher than mice received vehicle injection (*P* > 0.05, MK vs. CNO, *P* < 0.01, Veh. vs. CNO, *n* = 8, 9, 7 mice, one-way ANOVA followed by Tukey’s test), indicating inhibition of MB facilitated the retrieval of fear memory that was learned under MK-801. Furthermore, while injection of MK-801 before test significantly reduced freezing behavior of the control group (Fig. 3i, *P* < 0.01, Group 1 Day 1 vs. Group 1 Day 2, *n* = 8 mice, two-way ANOVA followed by Sidak’s test), mice both trained and tested under CNO showed robust freezing under MK-801 (Fig. 3i, *P* > 0.05, Group 2 Day 1 vs. Group 2 Day 2, *n* = 9 mice, two-way ANOVA followed by Sidak’s test), suggesting MK-801 could also recover the conditioned fear encoded under the CNO state. We also recorded locomotor activity and foot-shock induced hyperactivity under CNO (Fig. 3j), the results showed that CNO had no effect on mice locomotor behavior which excluded the possibility that the increased freezing with the presence of CNO was due to some behavioral side effects of the drug (*P* > 0.05, no shock Veh. vs. no shock CNO, *P* > 0.05, shock Veh. vs. shock CNO, *n* = 17, 17 mice, two-way ANOVA followed by Sidak’s test). Mice were all sacrificed after experiments and the expression of virus were checked. Only animals with virus expression restricted to the MB were included in our data. Taken together, our results suggested that the state-dependent fear memory induced by MK-801 was achieved via inhibition of MB neurons. Although, MK-801 also inhibited the neural activity in the hippocampus and cortex, the fact that inhibition of MB neurons alone allows the successful recall of the fear memory encoded under MK-801 suggested that MB is one of the key regulatory nucleuses involved in MK-801 induced state-dependent memory.

### Inhibition of MB neurons induces a delta oscillatory state in awake mice

**Figure 4:**
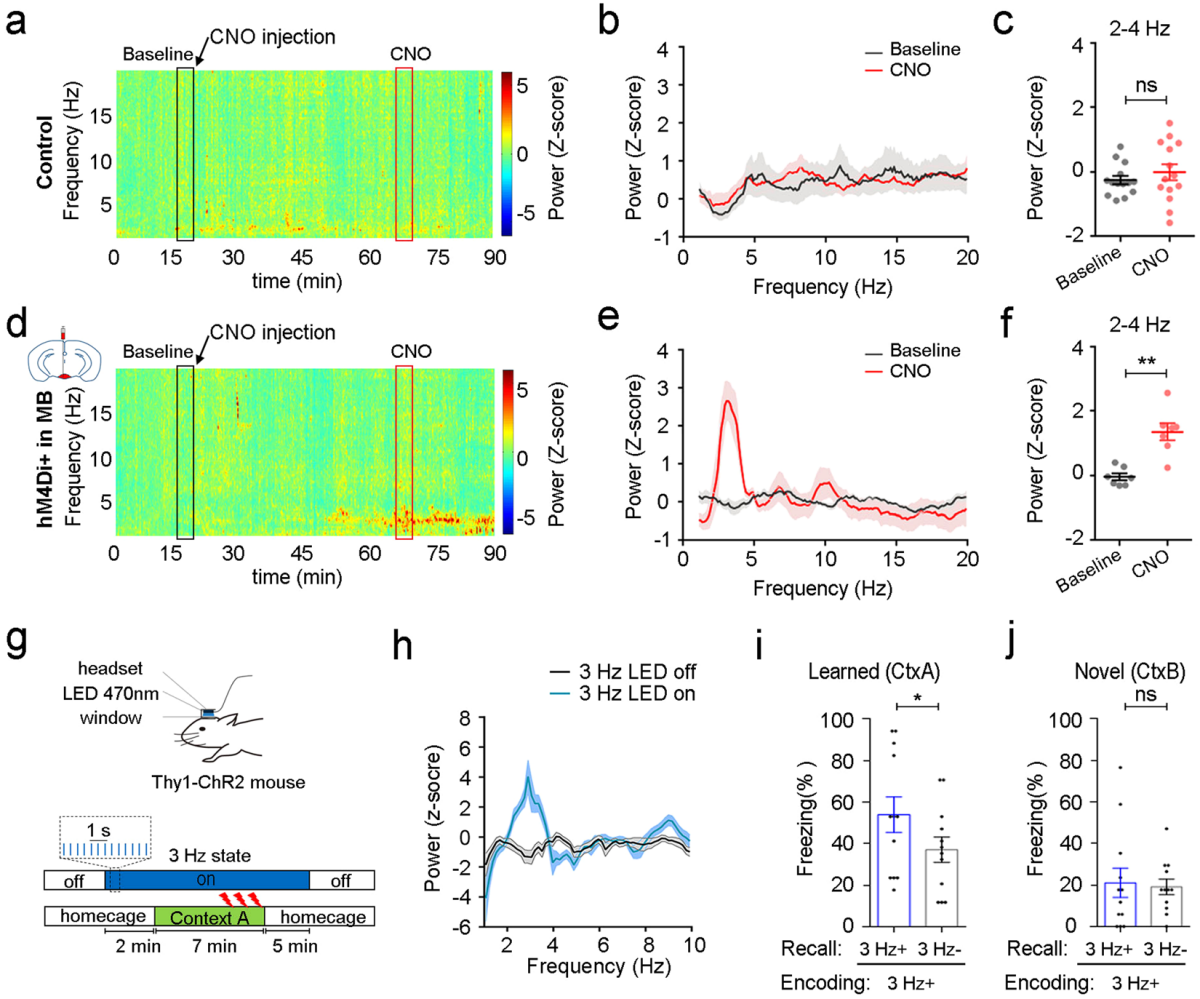
Inhibition of the mammillary body in awake animals increases the power of cortical delta waves which alone could induce state-dependent fear. (**a**,**d**) Average spectrogram of cortical LFPs in the control mice (**a**, *n* = 14 sites from 7 mice) and mice with hM4D-expression in the MB (**d**, *n* = 7 sites from 4 mice) under the normal and the CNO (2 mg/kg) states. Black/red box (5 min) labels the signal used as baseline/CNO onboard in the following statistical analysis. (**b**,**e**) Average power spectrum of cortical LFPs in the control mice (**b**, *n* = 14 sites from 7 mice) and mice with hM4D-expression in the MB (**e**, *n* = 7 sites from 4 mice) during the baseline and the CNO onboard periods. Shadow areas indicate SEM. (**c**,**f**) Quantification of the average power of delta band (2–4 Hz) during the baseline and the CNO onboard periods in the control mice (**c**, *n* = 14 sites from 7 mice, paired *t* test) and hM4D-expression mice (**f**, *n* = 7 sites from 4 mice, paired *t* test). (**g**) Experimental design for optogenetical induction of 3 Hz oscillatory state in the neocortex of Thy1-ChR2-EYFP mice and paradigm for fear conditioning (2 s, 0.8 mA, 3 foot shocks) under the delta-oscillatory state induced by LED stimulation. (**h**) Average power spectrum of cortical LFPs in head-fixed mice without and with blue light (470 nm, 3 Hz, 1 ms) on the RSC (*n* = 76 trials from 4 mice). Shadow areas indicate the trial-to-trial SEM. (**i**) Thy1 mice, which were trained in the pre-conditioned 3 Hz state (3 Hz, LED on the RSC, VIS, PTLp and SSp), showed increased freezing during 3 min light-on epochs when tested in the conditioning context (*n* = 6 mice, paired *t* test). (**j**) Mice did not show light-associated increase of freezing behavior when tested in a novel context (*n* = 6 mice, paired *t* test). ns: *P* > 0.05, *P < 0.05, **P < 0.01. Data presented as mean ± SEM.

Previous works have shown that thalamus plays an important role in the generation and modulation of brain oscillations^38–40^. Computational models have also shown that reducing the input to the thalamus would drive the synchrony between cortical and thalamic neurons and generate delta oscillations. Interestingly, by virus tracing, we found that MB neurons project intensively to the thalamus nucleus (Supplementary Fig. 2a,b). The major efferent projection from the MB is to the anterior thalamus nucleus (ATN), including the anterodorsal (AD), anteroventral (AV) and anteromedial (AM) thalamic nucleus, via the mammillothalamic tract (MTT), which is consistent with reported results^41^. Further tracing of neurons in the ATN showed dense projections in multiple cortical areas, including the medial prefrontal cortex (mPFC), anterior cingulate cortex (ACC) and retrosplenial cortex (RSC) (Supplementary Fig. 2c–e). Since activity of thalamus neurons plays an important role in cortical oscillations^38,42,43^ and MB shows strong projection to the ATN, which led us wondering if inhibition of MB neurons could also shift the brain rhythm to the high delta state just like the one induced by MK-801. To test this hypothesis, we performed cortical LFP recordings in head-fixed WT mice and mice with hM4D-expression in MB neurons under CNO (Fig. 4a–f and Supplementary Fig. 3a,b). While CNO had no effect on low-frequency cortical oscillations in WT mice (Fig. 4a–c, *t*_13_ = 0.7705, *P* = 0.4548, *n* = 14 sites from 7 mice, paired *t* test, ACC and RSC), CNO injection in hM4D-expression mice significantly increased the power of delta oscillations (Fig. 4d–f, *t*_6_ = 6.003, *P* = 0.0010, *n* = 7 sites from 4 mice, paired *t* test, ACC and RSC). The similar pattern of cortical oscillations induced by both MK-801 and MB silencing provided further evidence, suggesting that they work through the same mechanism in generating state-dependent learning and that delta waves may contribute to the state-dependent fear induced by MK-801.

### Delta oscillations underlies the state-dependent memory induced by MK-801

To test if delta oscillations in neocortex were directly associated with state-dependent memory, we applied blue-light-LED (470 nm, 3 Hz, 1 ms pulse) stimulation to the dorsal neocortex of Thy1-ChR2-EYFP mice, including the retrosplenial cortex (RSC), visual cortex (VIS), posterior parietal association areas (PLTp) and somatosensory cortex (SS) to induce the delta-oscillation state (Fig. 4g). Consistent with previous report^44^, LED flash activated layer 5 neurons to generate low-frequency oscillations in awake mice and increase the power of LFPs accordingly (Fig. 4h and Supplementary Fig. 3c,d). To simulate training under the delta-oscillation state, LED stimulation was applied 2 min before conditioning and remained for another 5 min after training. Fear memory in the training context (Ctx A) during both light-on and light-off epochs was tested. Freezing level during light-on epochs was significantly higher compared to that during light-off epochs (Fig. 4i, *t*_11_ = 2.692, *P* = 0.0210, *n = *12 from 6 mice, paired *t* test), which indicated light induced recall of fear memory. To rule out the possibility that the post-training LED stimulation was treated as a cue for the fear memory, the same group of mice was further tested in a novel context (Ctx B) and freezing level was also measured during both light-on and light off epochs (Fig. 4j). Despite the fact that mice underwent the same experimental procedure, light did not induce freezing when tested in a novel context (*t*_11_ = 0.2919, *P* = 0.7758, *n* = 12 from 6 mice, paired *t* test). This result indicated the increased freezing in the training context was not caused by any non-specific effects of the optical stimulation.

**Figure 5:**
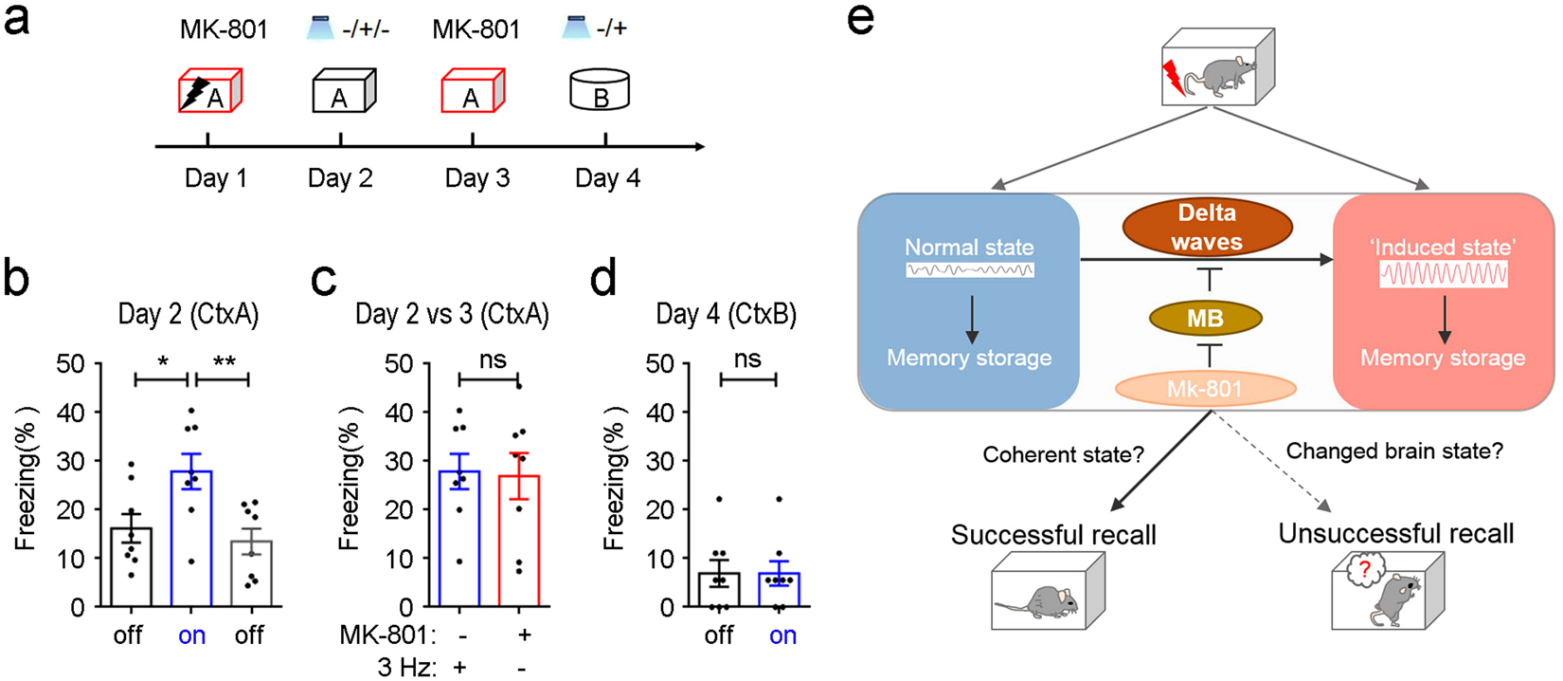
Memory formed under MK-801 is able to be retrieved by inducing delta oscillations in neocortex. (**a**) Experimental design for testing memory transfer between the MK-801-induced state and state induced by 3 Hz stimulation. Thy1-ChR2 mice were trained (2 s, 0.6 mA, 3 foot shocks) under MK-801 and memory test was performed under different conditions. (**b**) Mice showed increased freezing during the 3-min-light-on epochs when tested in the conditioning context (*n* = 8 mice, one-way ANOVA followed by Tukey’s test). (**c**) Freezing behavior during the light-on epochs was as robust as that tested under MK-801 (*n* = 8 mice, paired *t* test). (**d**) Mice did not show light-associated increase of freezing behavior when tested in a novel context (*n*8 mice, paired *t* test). ns: *P* > 0.05, *P < 0.05, **P < 0.01. Data presented as mean ± SEM. (**e**) Schema illustrates the MB as a key regulator of brain state for state-dependent memory through regulating brain oscillations.

Finally, to test if delta waves induced by MK-801 were directly linked to the regulation of state-dependent memory, we enforced delta oscillations in mice following the fear memory acquired under MK-801. Thy1-ChR2 mice were trained under MK-801 and tested under both the normal and delta oscillatory states induced by LED stimulation (Fig. 5a). Mice expressed significantly increased freezing after the induction of delta oscillations (Fig. 5b, *P* < 0.05, light-off 1 vs. light-on, *P* < 0.01, light-on vs. light-off 2, *n* = 8 mice, one-way ANOVA followed by Tukey’s test). Such LED-induced freezing on day 2 was similar to that on day 3 when the mice were test under MK-801 (Fig. 5c, *t*_7_ = 0.2367, *P* = 0.8196, *n* = 8 mice, paired *t* test). Whereas, on day 4, when the mice were tested in a neutral context, LED-induced delta waves in neocortex did not induce freezing (Fig. 5d, *t*_7_ = 1.311e-0.007, *P* > 0.9999, *n* = 8 mice, paired *t* test), indicating the LED-induced delta waves will not directly lead to fear behavior. Thereby, the LED-induced delta oscillatory state was able to allow the retrieval of the context-associated fear memory encoded during the period when MK-801 was onboard (Fig. 5e).

## Discussion

The generation of state-dependent memory by different kinds of centrally acting drugs has been studied extensive, yet its mechanism remains to be investigated. In the current study, we reported that the amnestic effect of MK-801 was actually a phenomenon of state-dependent memory which achieved via MB inhibition. MK-801 suppressed the activity of MB neurons and induced strong delta waves in awake mice, generating an alternative state for memory encoding and recall. Moreover, memory learned under MK-801 could be recalled by artificially reconstructing the cortical delta-oscillatory pattern. Thus, brain rhythm is one of the key components of brain state, which determines the internal framework for memory encoding and retrieval. Our experiments further strengthened the idea that the brain state was regulated by the thalamic nucleus, such as the MB and the change of brain state might account for the fluctuation of successful memory retrieval.

Although the role of NMDA receptor in synaptic plasticity has been well established and the low-dose effect of NMDA receptor blocker MK-801 in this study appeared to be surprising, we noticed that similar effects of NMDA receptor antagonists on memory have emerged in some early reports in rodent model of food-rewarded lever-pressing task^3^ and passive avoidance task^10,45^, suggesting the state-dependent memory phenomenon may contribute to some failures of memory recall induced by MK-801.

Different states of the brain are characterized by distinct cortical oscillations and are strongly associated with behavioral states^11,46^. Alpha waves are increased when eyes are closed. Theta bands are engaged in moving and in rapid-eye-movement (REM) sleep. High frequency bands such as gamma oscillations have been linked to cognitive process, including attention and imagination. Studies suggested that the dominant oscillatory activity in neocortex might affect the range and the size of neuron population for efficient communication^11,12,47^. Thus, alternation of oscillatory rhythms could change the way of information processing and memory coding in the brain. In this study, we observed that the potentiated delta waves were associated with the administration of MK-801 and MB inhibition, and provided evidence suggesting that memory retrieval is regulated by the oscillatory-activity defined brain state. Thalamus is a crucial regulator of brain oscillations^38,42^. Experimental evidences have also shown that thalamus inhibition could drive cortical delta oscillations^43,48^. Since thalamus is the major efferent of MB^41^, we speculated that suppressed thalamus activity might account for MK-801 induced cortical delta waves.

Evidence has shown that MB is critical for memory retrieval, as atrophy of which is associated with memory defect in MB damaged patients with alchoholic Korsakoff syndrome^49^ and Alzheimer’s disease^50^. Complete MB lesion in animal model impaired memory in the water maze task^51^. However, human studies on amnesia following MB damage are not very consistent. Anterograde or retrograde amnesia after MB atrophy was reported in several case-reports^35,52^, but MB volume shrinkage also occurred in alcoholics without amnesia. In some cases, Korsarkoff syndrome patients with diagnosed amnesia symptom did not show any visible MB atrophy^53,54^. In this study, we found chemogenetically silencing of MB during learning impaired fear response to the conditioning context. Contrary to the behavioral results in MB lesion animals^51,55^, fear memory encoded under the MB silencing state could be facilitated when MB was inhibited again, suggesting an alternative explanation for the role of MB as a regulator of brain state during memory processing.

In summary, we found that cortical delta oscillations define a unique brain state for memory encoding and retrieval. Such brain state has a strong effect on memory processing, as the memory encoded in either the natural or the delta oscillatory state could only be retrieved when the same state is reestablished. Furthermore, MB is an important regulatory nucleus, governing the brain state transitions. MK-801 strongly inhibits the activity of MB neurons, thereby, shifts the brain to a delta-oscillation state, in which memory encoded is inaccessible in the normal state. These data implicated that brain oscillations could account for the fluctuation of successful memory retrieval.

## Methods

### Subjects

The ethics committee of Tsinghua University approved the experimental protocols used in this study (Assurance Identification Number: 15-GJS1). Animal care was in accordance with the Institutional guidelines of Tsinghua University. Protocols are proved by IACUC in Tsinghua University. All the animals were socially housed in a 12 h/10 h (7 am–7 pm) light/dark cycle, with food and water adalibitum. The Thy1-ChR2-EYFP (007612) mice were distributed from Jackson Laboratories. C57BL/6 wild-type mice were obtained from Beijing Vital River Laboratory Animal Technology Co., Ltd.

### Virus preparation

AAV8-hSyn-hM4D(Gi)-mCherry (Viral titre: 8.2 × 10^12^ genome copy ml^−1^), AAV8-hSyn-GCaMP6f (Viral titre: 1.6 × 10^13^ genome copy ml^−1^), AAV8-hSyn-EGFP (Viral titre: 1.2 × 10^13^ genome copy ml^−1^) and AAV8-CMV-EGFP (Viral titre: 1 × 10^13^ genome copy ml^−1^) were bought from Obio Technology (Shanghai) Co., Ltd.

### Drug injections

MK-801 (Sigma-Aldrich) was made in a stock solution of 5 mg/ml in ethanol and diluted in PBS to desired concentrations. MK-801 was injected (i.p.) at the dose of 0.1 mg/kg (behavioral experiment which used the dose of 0.2 mg/kg was pointed out in Fig. 1) 30 min before contextual fear conditioning or memory retrieval. Clozapine-*N*-oxide (CNO; Sigma-Aldrich) was made in a solution of 0.6 mg/ml in PBS and injected (i.p.) at the dose of 2 mg/kg 45 min before contextual fear conditioning or memory retrieval. PBS was used as the control vehicle.

### Surgery

All the surgeries were performed under stereotaxic guidance. Data used were based on histological criteria including virus injection site, virus expression and optic fiber implantation site. Only the mice with the right virus injection site and fiber implantation were included. After surgery, mice were allowed to recover for at least 2 weeks before experiments. During the surgery, mice were anesthetized with 5% isoflurane and placed into a stereotaxic frame and maintained anesthetized with 1.5% isoflurane.

For the pharmacogenetic, fiber recording and tracing experiments, 0.5 μl AAV8-hSyn-hM4D(Gi)-mCherry, AAV8-hSyn-EGFP, AAV8-hSyn-GCaMP6f or AAV8-CMV-EGFP was injected to the target site (speed: 0.08 μl/min), based on the experimental design. The virus was injected using a 10 μl Hamilton syringe. A microsyringe pump and its controller (KD Scientific) were used to control the volume and speed of the injection. The needle was slowly lowered to the target site and remained for 5 min before the beginning of the injection. After the injection, the needle stayed for another five minutes before it was slowly withdrawn.

For the fiber recording experiment, after the injection of virus expressing GCaMP6f into the target region, optic fiber was inserted into the MB (0.2 mm above the virus injection site). Dental cement was applied to secure the optical fiber implant.

The stereotaxic coordinates were described in below. MB: AP, −2.8 mm; ML, 0.0 mm; DV, 6.2 mm from skull; ATN: AP, −0.8 mm; ML, ± 0.7 mm; DV, 4.5 mm from skull.

### Contextual fear conditioning task

All the behavior experiments were performed in the TSE system. All the fear conditioning experiments were done in context A (Ctx A), which was a 20 × 20 × 39.5 cm square chamber with metal gridded floor and 75% ethanol. Context B (Ctx B) was an unscented chamber 30 × 30 × 24.5 cm with plastic floor and dim lighting. Training consists of a 3-min exposure of mice to the conditioning box (Multi-conditioning system, TSE system) followed by 3 foot shocks: 2 s, 0.8 mA (0.6 mA in Fig. 5), constant current, 20 s interval between each shock. Ten seconds after the last shock, mice were placed back to their homecages. Memory test was performed by re-exposing the mice to the conditioning context for 3 min. Freezing, defined as a lack of movement except for the heart beat and respiration associated with a crouching posture, was recorded by a video and rated every 10 s by two blinded observers (unaware of the experimental conditions) during 3 min (a total of 18 sampling intervals). The number of observations indicating freezing obtained as a mean from both observers was expressed as a percentage of the total number of the observations.

For the fear conditioning in the Thy1-ChR2 mice, mice received cranial window implantation and C shaped headsets were glued to the window with dental cement to hold LED (5050 model, 470 nm, 5 mm × 5 mm × 1.6 mm, 3.3 V, 3.6 mW). LED were directly connected to LED single controller (Arduino DUE board) together with a toggle switch, if necessary, to manually control. Connected wire (d = 0.45 mm) is soft and slender to avoid affecting the normal movement of the mice. To prevent the direct driving of body movement, the part of the window over the motor area was blotted out by a black shutter. Mice were allowed to recover for 1–2 weeks before the subsequent experiments.

Behavioral design in Fig. 4: for the training section, mice received LED (470 nm, 3 Hz, 1 ms) stimulation on the dorsal part of the cortex to induce delta waves. The LED stimulation was started 2 minutes before conditioning when the mice were still in their homecages and remained on for another 5 minutes after the mice returned to their homecages. During the contextual fear conditioning, mice were hold in context A for 3 minutes and received three foot shocks (2 s, 0.8 mA, constant current, 2 min interval between each shock). During test, mice were allowed to explore the chamber (Ctx A) for 12 min. The 12 min session was divided into four 3-min epochs, with the first and third epochs as the light-off epochs, and the second and fourth epochs as the light-on epochs. The order of light-on and light-off epochs for different mice were randomly shuffled. For the test in a novel context, mice were tested in Ctx B at 24 hours after tested in the Ctx A with all other procedures unchanged.

### Fiber Photometry and data analysis

The fiber photometry system were bought from Thinker Tech Nanjing Biotech Limited Co. Excitation light from a 488-nm semiconductor laser (Coherent, Inc. OBIS 488 LS, tunable power up to 60 mW) was reflected by a dichroic mirror with a 452–490 nm reflection band and a 505–800 nm transmission band (Thorlabs, Inc. MD498), and then coupled to a fiber (Thorlabs, Inc., 200 μm in diameter and 0.37 in numerical aperture) by an objective (JiangNan, Inc. 20×, NA 0.4). The emission fluorescence was collected with the same optical fiber and then detected by a high sensitive photomultiplier tube (PMT) (Hamamatsu, Inc. H10720-210) after filtering by a GFP band pass emission filter (Thorlabs, Inc. MF525-39). The laser intensity at the interface between the fiber tip and the animal was adjusted to around 50 μW to minimize bleaching. Signals were collected at a sampling frequency of 100 Hz. An amplifier (C7319, Hamamatsu) was used to convert the photomultiplier tube current output to voltage signals, which was further filtered through a low-pass filter (35 Hz cut-off; Brownlee 440). The analogue voltage signals were digitalized and collected using Fiber Photometry software (Thinker Tech Nanjing Biotech Limited Co.) and further analyzed using Matlab.

The fiber photometry recording was performed on freely moving mice and the photometry data were exported to Excel and then MATLAB Mat files for further analysis.

For testing MB activity in response to MK-801 (0.1 mg/kg, i.p.), we segmented the data based on difference between baseline and drug onboard. We derived the values of fluorescence change (Δ*F*/*F*_0_) by calculating (*F* − *F*_0_)*/*F_0_, where *F*_0_ is the baseline fluorescence signal averaged over a 1-sec-long control time window around each time point. Δ*F*/*F*_0_ values were presented with plots and two parameters were quantified to analyzing the activity level. We derived the values of area under curve (AUC) by calculating the mean value of Δ*F*/*F*_0_. To quantify the values of activity frequency, we calculated all the time fragment containing points above a particular threshold (Δ*F*/*F*_0_ > 0.06, which was far beyond the noise level) and lasted for at least 10 ms. Then we sorted every positive fragment to 1, 2 or more activity events by the length of the fragment (event count = 1 + length of the fragment/100 ms, rounding down to the nearest whole number).

For the statistics of calcium transients in Fig. 2d, each point represents a averaged signal of 5 min. Drug injection time point was set as 0. Signals used as baseline and drugs composed of the averaged signals recorded from −5 min to 0 min and 30 min to 35 min separately. These data were normalized to the mean signals recorded from −10 min to −5 min before further statistics analysis.

### Electrophysiological recording and data analysis

Local field potentials (LFPs) were recorded with a custom-made electrode (Ni-Cr alloy wires, California Fine Wire Company, CFW 100188, 35 μm), we recorded one or two cortical regions (RSC, ACC, ~500 μm deep) in each head-fixed aroused mouse with channel electrodes sharing one reference (Ni-Cr alloy wires), which was placed in saline that covered the cortex (at a position ~1 mm above the RSC surface). Signals were amplified by AM-1800 amplifier and then digitized by Digidata-1440. The notch function is turned on to reduce the line noise and the distorted waveband (47–53 Hz) data was excluded in following data analysis. LFPs were band-pass filtered between 1–1000 Hz and sampled at 1 kHz. Mice were adapted to the recording apparatus for 30 min in 4 continuous days before recording.

All the data analyses were performed using custom-written Matlab (MathWorks) scripts. In the spectrogram and spectrum analyses, the power spectrums of LFPs were computed using the multi-taper estimation (3–5 tapers (TW = 3, K = 5)) method in Matlab with the chronux package (http://chronux.org). The power was normalized by the mean power across all frequency bands: standard deviation and the mean value of 0–5 min LFP signals after CNO (2 mg/kg) or MK-801 (0.1 mg/kg) injection (the injection time point was set as 0 min) were applied to normalize the whole length data (z-score) in each frequency (1–70 Hz). Spectrums of baseline (−5–0 min) and drug on board (45–50 min for CNO, 30–35 min for MK-801) were the averaged data during the 5 minutes of the normalized spectrogram. Filtered sample traces were obtained using zero-phase-distortion IIR Butterworth filters (Matlab filter design tool box, filter parameters: Fstop1 = 1.5 Hz, Fpass = 2 Hz, Fpass2 = 4 Hz, Fstop2 = 4.3 Hz. Astop1 = 25; Apass = 0.5; Astop2 = 25).

To record the LFPs induced by blue light (3 Hz, 470 nm) in the cortex of Thy1-ChR2 mouse, electrophysiological recording with light stimulation was done as described above. For each 25-second trial, the blue light (470 nm, ~5 mW, 3 Hz, 1 ms) was delivered for 4 s via the optic fiber attached to the laser source (Fiblaser Technology, Co. Ltd). Electrode was placed in the RSC (500 μm deep). Optic fiber (200 μm, diameter) was placed above the cortical surface (1 cm).

To analyze the LFP spectrum data, the power was normalized by the mean power across all the frequency bands: standard deviation and the mean value of the first second of each trial.

### Western blotting

For western blotting experiments, mice aged 3–4 months were decapitated and brains were quickly removed and transferred to ice. Target brain regions were obtained under stereoscope and lysed in lysis buffer (50 Mm Tris-HCL; 150 mM NaCl; 1% Triton X-100; 10% glycerol; 0.5% BSA; pH 7.4). 1× protease inhibitor cocktail (Roche) were added to lysis buffer before use. The concentrations of samples were determined using the bicinchoninic acid (BCA) assay (Thermo) and were analyzed directly via sodium dodecyl sulfate-polyacrylamide gel electrophoresis (SDS-PAGE). The proteins were then transferred to polyvinylidene difluoride membranes. The membranes were incubated in the following primary antibodies overnight at 4 °C (anti-NR1, 1:1000, #05–432^56^, Millipore, given by Yelin Chen lab; anti-GAPDH, 1:3000, Abmart, M20028^57^). The membranes were then incubated in an anti-rabbit or anti-mouse secondary antibody conjugated to horseradish peroxidase (1:5000, TDYbio S001 and S004). Immunoblots were analyzed with ImageJ.

### Confocal imaging and histological analysis

To test the neuronal activity using c-Fos immunostaining, 2 hours after drug (MK-801, 0.1 mg/kg; CNO, 2 mg/kg) injection (i.p.), mice were transcardially perfused with 4% PFA, followed by 24 h post-fixation in the same solution and then were changed to 20% sucrose solution for another 24 h. Free-floating 50-µm coronal sections were prepared using a vibratome. Sections were incubated in the blocking solution (0.1% Triton X 100, 1% Bovine Serum Albumin in PBS) with anti-c-Fos rabbit primary antibody (CST, 1:500 dilution for 12 h at 4 °C). After series of PBS washes sections were stained using Alexa Fluor 647 goat anti-rabbit secondary antibody (life technology, 1:500 dilution) and DAPI (Beyotime, 1:10000 dilution) diluted in PBS for 45 min at 37 °C. After a series of PBS washes, sections were mounted on slides.

Images were obtained either in a confocal laser-scanning microscopy (Olympus) with an UltraView Vox system (PerkinElmer) with either 10× or 20× air immersion objectives or in Zeiss Axio Scan Z1. A semi-automated method was used to quantify viral infection and c-Fos expression in the confocal images of brain slices. Equally thresholded images were subjected to multi particle analysis (NIH ImageJ). Intensity values of area of region of interest (ROI) were obtained from raw images by using Multi Measure tool. The number of c-Fos and hM4D double positive neurons in MB and the number of c-Fos positive neurons in DG were counted manually. All counting was performed by a blinded experimenter who was unaware of the experimental conditions. The number of c-Fos positive neurons was further normalized by the area of the region before statistical analyses were performed. Each point in the panel of quantification data represent the average c-Fos expression level of three slices from one mouse.

### Statistical analysis

GraphPad Prism version 6.00 (GraphPad Software, La Jolla, California, USA) was used for statistical analyses. Statistical significance was assessed by two-tailed paired Student’s *t*-tests, two-tailed unpaired Student’s *t*-tests, one-way ANOVA, or two-way ANOVA where appropriate. Significant effects or interactions were followed up with post hoc tests with the use of either Tukey’s or Sidak’s multiple comparison test. Significance levels were set to *P* = 0.05. Significance for comparisons: *P < 0.05; **P < 0.01; ***P < 0.001.

## Electronic supplementary material

Supplementary information

## Data Availability

All data is available by addressing to J.S.G. (email: guanjs@shanghaitech.edu.cn) or J.J. (email: j_jiang08@qq.com).
