# Supplementary material for: Mammillary body regulates state-dependent fear by alternating cortical oscillations
Source: Sci Rep. 2018 Sep 7;8:13471. doi: 10.1038/s41598-018-31622-z (PMC6128928; doi:10.1038/s41598-018-31622-z)
Supplement: Supplementary file 1 — Supplementary information [file 41598_2018_31622_MOESM1_ESM.pdf]

Mammillary body regulates state-dependent fear by alternating cortical oscillations

Jun Jiang[1, 3#], **Guang-Yu Wang**[1, 3#], **Wenhan Luo**[2, 4, 1#], **Hong Xie**[1], **Ji-Song Guan**[3, 1*]

[1]MOE Key Laboratory of Protein Sciences, IDG/McGovern institute for Brain Research at Tsinghua, School of Life Sciences, Tsinghua University, Beijing 100084, China

[2]Peking-Tsinghua Center for Life Sciences, Beijing 100871, China

[3]School of Life Science and Technology, ShanghaiTech University, Shanghai 201210, China

[4]Academy for Advanced Interdisciplinary Studies, Peking University, Beijing 100871, China

*Correspondence: guanjs@shanghaitech.edu.cn

[#]These authors contribute equally to this paper

Supplementary information

Supplementary Figure 1. Related to Figure 2.

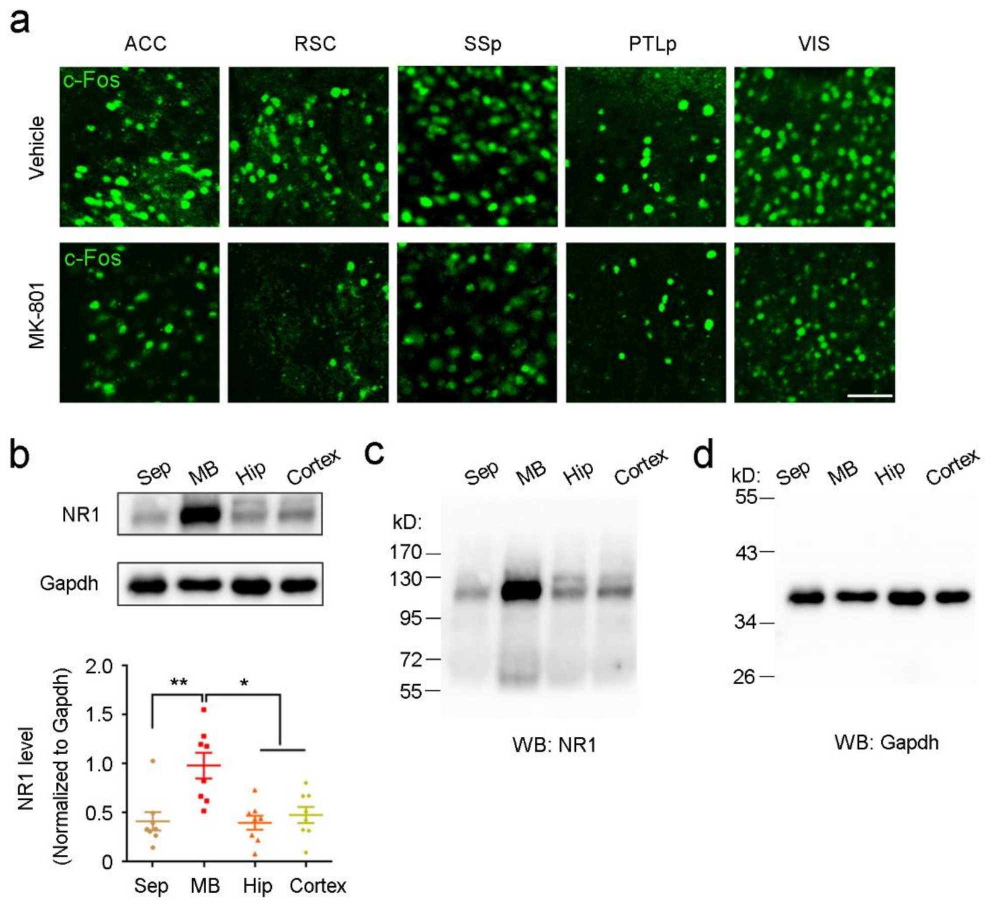

(**a**) Representative images of c-Fos expression in different cortical regions. Green, c-Fos. Scale bar: 50 µm. (**b**) Representative western blot for the expression of NR1 subunit in different brain regions and statistical results (*n* = 8 from 4 mice, technical repeat: 2; one-way ANOVA followed by Tukey's test). These two gels were cropped from different parts of the same gel. (**c** and **d**) Uncropped blot of NR1 (c) and Gapdh (d) in panel **b**. Proteins in SDS-PAGE were transferred to polyvinylidene difluoride membrane. The memory was cut into two halves at the marker of 55 kD and then incubated with different primary antibodies (top, anti-NR1; bottom, anti-GAPDH).

Supplementary Figure 2. Projections of the MB. Related to Figure 4.

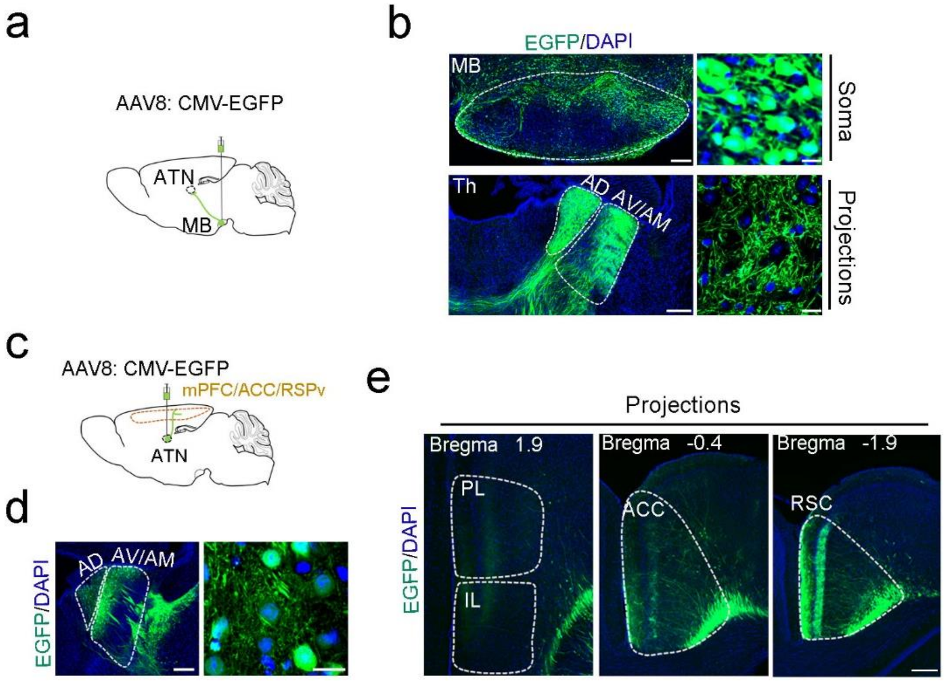

(**a** and **b**) Design (**a**) and representative images (**b**) of AAV8: CMV-EGFP labeled input neurons in the MB and the axonal projections detected in the ATN, including the AD and AV/AM. Scale bar: left, 200 μm; right, 20 μm. (**c**) Schema illustrating experimental design used for anterograde tracing from the ATN. (**d**) Example images showing virus expression in the ATN. left, 200 μm; right, 20 μm. (**e**) Representative figures showing axonal projections from the ATN that detected in the IL/PL, ACC and RSC. Scale bar: 200 μm. ATN, anterior thalamic nuclei. Th, thalamus. AD, anterodorsal thalamus. AV, anteroventral thalamus. AM, anteromedial thalamus. IL, infralimbic cortex. PL, prelimbic cortex. ACC, anterior cingulate cortex. RSC, retrosplenial cortex.

Supplementary Figure 3. Distinct modulations of cortical oscillations. Related to Figure 4.

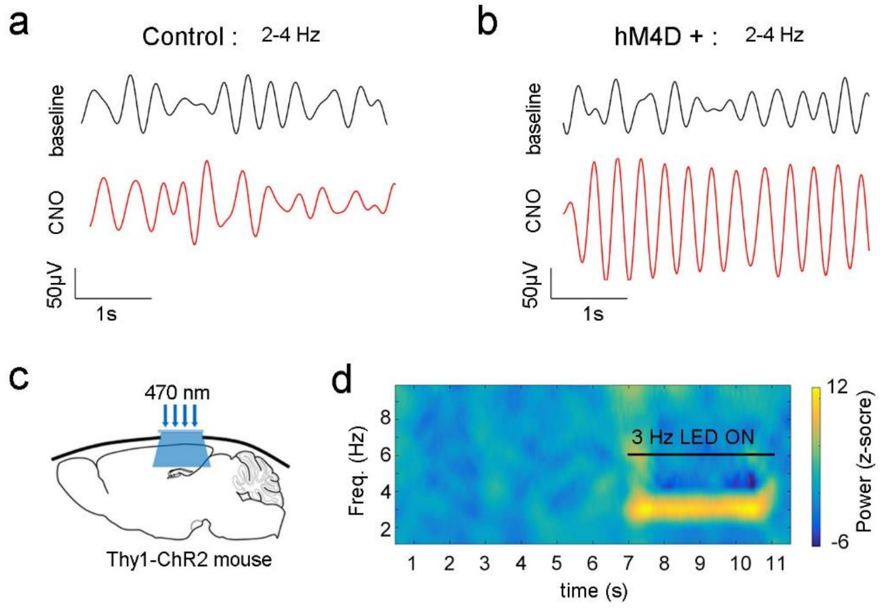

(**a** and **b**) One example of filtered (2-4 Hz) cortical LFP traces in the control mice (**a**) and mice with hM4D-expression in the MB (**b**) during the basal (baseline) and CNO onboard (CNO) conditions. The amplitude was strongly increased after CNO injection in the hM4D-expression mice but not the control mice. (**c**) Design for the induction of delta oscillations in cortex using 3 Hz-blue-light (LED, 470nm, 1 ms) stimulation. (**d**) One example spectrogram of cortical LFPs during the 3 Hz light-on and light-off epoch. Black line indicates light-on period.
